# New 5-Aryl-Substituted 2-Aminobenzamide-Type HDAC Inhibitors with a Diketopiperazine Group and Their Ameliorating Effects on Ischemia-Induced Neuronal Cell Death

**DOI:** 10.1038/s41598-018-19664-9

**Published:** 2018-01-23

**Authors:** Yoshiyuki Hirata, Tsutomu Sasaki, Hideaki Kanki, Chi-Jing Choong, Kumiko Nishiyama, Genki Kubo, Ayana Hotei, Masahiko Taniguchi, Hideki Mochizuki, Shinichi Uesato

**Affiliations:** 1 Department of Life Science and Biotechnology, Faculty of Chemistry, Materials and Bioengineering, Kansai University, 3-3-35, Yamate-cho, Suita, Osaka 564-8680 Japan; 2 Osaka University of Pharmaceutical Sciences, 4-20-1 Nasahara, Takatsuki, Osaka 569-1094 Japan; 3 Department of Neurology, Graduate School of Medicine, Osaka University, Yamadaoka 2-2, Suita, Osaka 565-0871 Japan

## Abstract

We previously synthesized new 5-thienyl-substituted 2-aminobenzamide-type HDAC1, 2 inhibitors with the (4-ethyl-2,3-dioxopiperazine-1-carboxamido) methyl group. K-560 (**1a**) protected against neuronal cell death in a Parkinson’s disease model by up-regulating the expression of XIAP. This finding prompted us to design new K-560-related compounds. We examined the structure activity relationship (SAR) for the neuronal protective effects of newly synthesized and known K-560 derivatives after cerebral ischemia. Among them, K-856 (**8**), containing the (4-methyl-2,5-dioxopiperazin-1-yl) methyl group, exhibited a promising neuronal survival activity. The SAR study strongly suggested that the attachment of a monocyclic 2,3- or 2,5-diketopiperazine group to the 2-amino-5-aryl (but not 2-nitro-5-aryl) scaffold is necessary for K-560-related compounds to exert a potent neuroprotective effect.

## Introduction

With a growing aging population, the number of individuals with neurological disorders, such as Alzheimer’s disease, Parkinson’s disease (PD), and ischemic stroke, is steadily increasing. It is challenging to design effective polypharmacological central nervous system (CNS) drugs because of the complex pathophysiological mechanisms of neurological disorders.

Histone deacetylase (HDAC) inhibitors reportedly ameliorated neuronal damage *via* pleiotropic effects, including anti-excitotoxicity, oxidative stress reduction, and inflammatory response suppression in *in vitro* and *in vivo* cerebral ischemia models^1^. However, the HDAC inhibitors used in these studies were non-specific, exemplified by valproic acid, trichostatin A (TSA), sodium butyrate, and SAHA (Vorinostat), and, thus, are associated with toxicities such as thrombocytopenia, nausea, fatigue, and QT prolongation^2,3^. These side effects underscore the need for more refined approaches to target HDAC subfamilies in order to reduce neuronal injuries. However, the type of isozyme inhibition that leads to neuroprotection remains unclear. Therefore, the selective targeting of HDAC isoforms with small molecules represents an attractive topic for the development of treatments for neurological disorders with few side effects.

**Figure 1:**
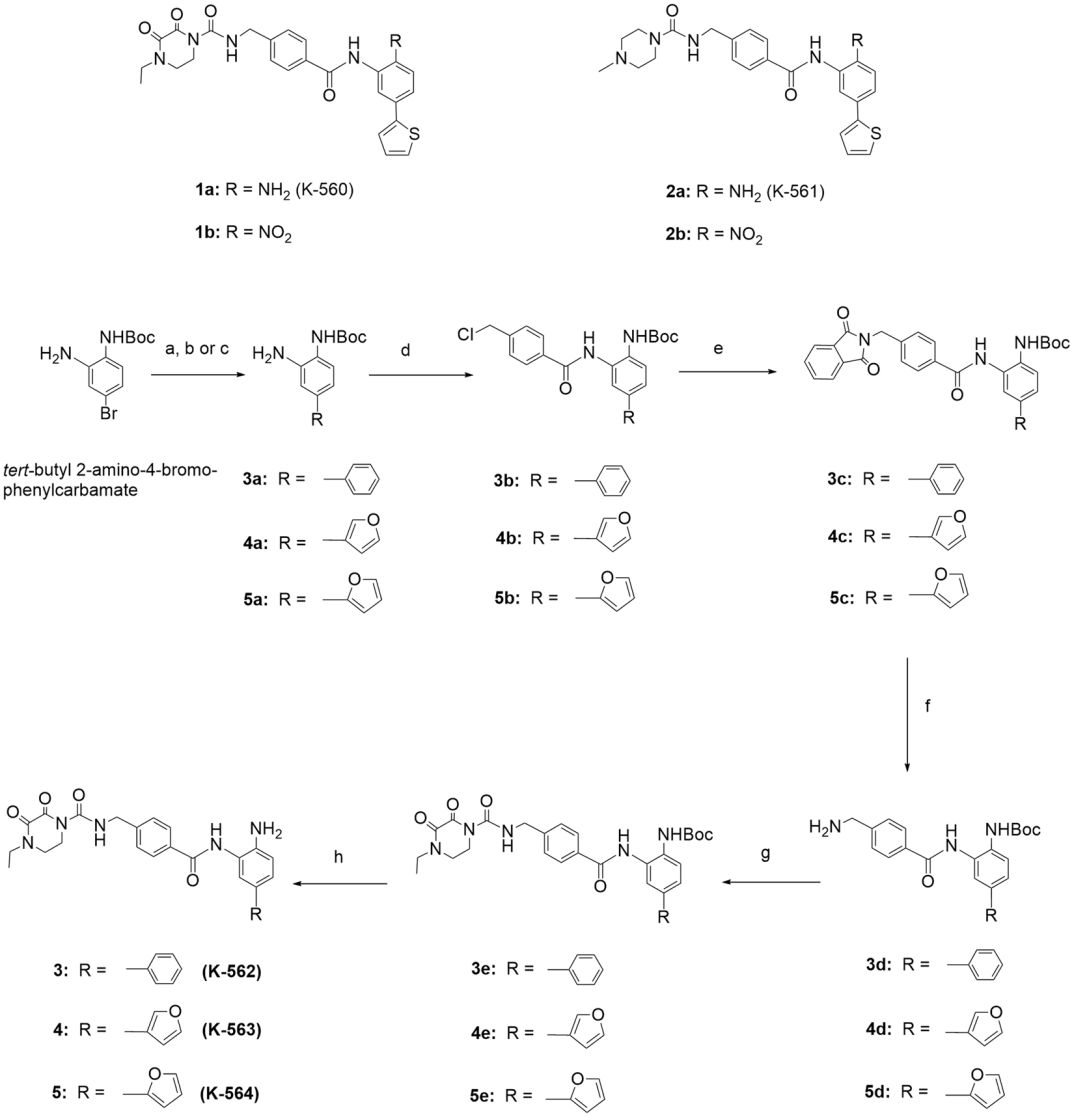
Structures of Known K560-Derivatives and Synthetic Routes of New K-560-Related Compounds **3**, **4** and **5**. Reagents and Conditions: (a) Pd(PPh_3_)_4_, phenylboronic acid, K_2_CO_3_, P(*o-*MeC_6_H_4_)_3_, DME, H_2_O; (b) Pd(PPh_3_)_4_, 3-furanboronic acid, K_2_CO_3_, P(*o-*MeC_6_H_4_)_3_, DME, H_2_O; (c) Pd(PPh_3_)_4_, 2-furanboronic acid, K_2_CO_3_, P(*o-*MeC_6_H_4_)_3_, DME, H_2_O; (d) *p-*(chloromethyl) benzoyl chloride, Et_3_N or Py, THF; (e) potassium phthalimide, KI, DMF; (f) NH_2_NH_2_·H_2_O, EtOH; (g) 4-ethyl-2,3-dioxo-1-piperazinecarbonyl chloride, Et_3_N, CH_2_Cl_2_; (h) TFA, CH_2_Cl_2_, then NaHCO_3_.

We previously synthesized 5-thienyl-substituted 2-aminobenzamide-type HDAC inhibitors, including K-560 (**1a**) possessing the (4-ethyl-2,3-dioxopiperazine-1-carboxamido) methyl group and K-561 (**2a**) having the (4-methylpiperazine-1-carboxamido) methyl group^4^ (Fig. 1). Compound **1a** inhibited HDAC1 and 2 selectively and suppressed the growth of cancer cells, similar to the same type of HDAC inhibitors^5–21^. However, **1a** averted the death of HCT116 human colorectal cancer cells by a mechanism involving activation of the survival signal-related proteins Akt/mammalian target of rapamycin (mTOR)/70-kDa ribosomal protein S6 kinase (p70S6K)^22,23^. This effect was also applied to neuronal cells, *i.e*., K-560 (**1a**) exerted protective effects against 1-methyl-4-phenylpyridinium ion/1-methyl-4-phenyl-1, 2, 5, 6-tetrahydropyridine (MPP^+^/MPTP)-induced neuronal death through the sustained expression of X-linked inhibitor of apoptosis protein (XIAP) *in vitro* and *in vivo* in a Parkinson’s disease model^23–25^. Since diketopiperazines themselves were reported to exert neuronal protective effects^26–28^, it is conceivable that the 4-ethyl-2,3-dioxo-1-piperazine moiety, at least partially, contributed to neuronal protection^23^. Therefore, we anticipate that HDAC1, 2 inhibitors possessing functional groups such as those in **1a**, have potential as therapeutic agents for neurodegenerative diseases with a new mechanism of action. These findings prompted us to synthesize the new K-560-related compounds as follows: K-562 (**3**), K-563 (**4**), and K-564 (**5**) with the (4-ethyl-2,3-dioxopiperazine-1-carboxamido) methyl group, and K-852 (**6**), K-854 (**7**) and K-856 (**8**) with a 2,3- or 2,5-diketopiperazinylmethyl group. Furthermore, to analyze the structure activity relationship (SAR) of the derivatives, OP-857 (**9**) and OP-858 (**10**) with a 3-oxopiperazinylmethyl group and OP-859 (**11**) with the 4-ethylpiperazinylmethyl group were prepared. We assessed the potency of these compounds as neuronal protective agents through measuring their toxicity degree to human neuroblastoma SH-SY5Ycells and cell viability in an *in vitro* model of cerebral ischemia, as well as their selectivity in HDAC1, 2, 3, 8 (Class I) and 6 (Class II) inhibition. We found that **8** with the 2,5-diketopiperazinylmethyl group exerted a promising neuronal protective effect, which was comparable to that of **1a**.

## Results

### Chemistry

**Figure 2:**
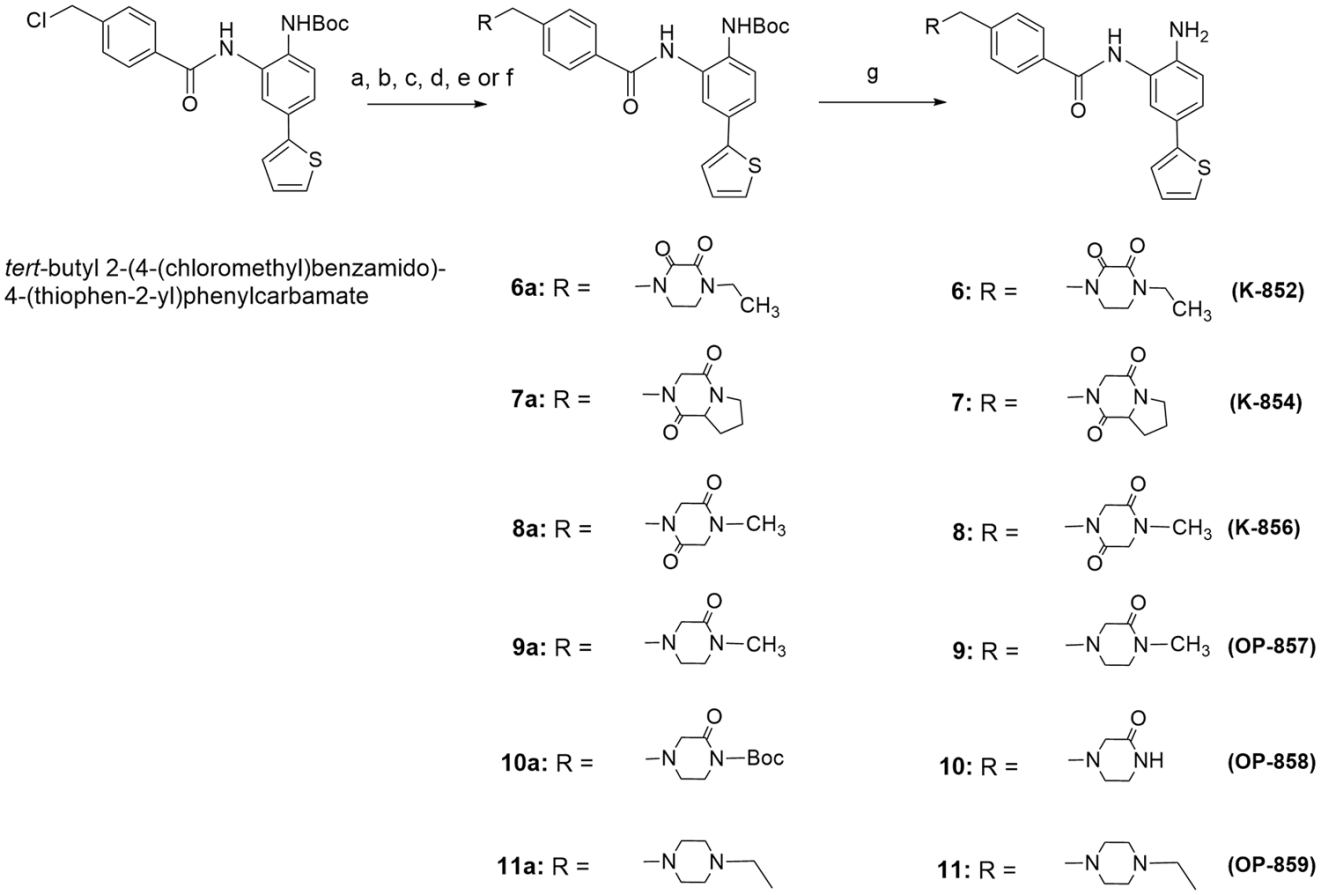
Synthetic Routes of New K-560-Related Compounds **6**, **7**, **8**, **9**, **10** and **11**. Reagents and conditions: (a) 4-ethyl-2,3-dioxo-1-piperazine, NaH, DMF; (b) cyclo-L-prolylglycine, NaH, DMF; (c) 1-methylpiperazine-2,5-dione, NaH, DMF; (d) 1-methyl-piperazin-2-one, NaH, THF; (e) *tert*-butyl 3-oxopiperazine-1-carboxylate, NaH, THF; (f) 1-ethylpiperazine, NaH, THF; (g) TFA, CH_2_Cl_2_, then NaHCO_3_.

In order to identify more potent compounds than K-560 (**1a**) for neuronal protection, we attempted to synthesize the following two types of compounds: K-560 analogues possessing a 5-phenyl (K-562 (**3**)), 5-(furan-3-yl) (K-563 (**4**)), or 5-(furan-2-yl) (K-564 (**5**)) group in place of the 5-(thien-2-yl) group in **1a**, and K-560 derivatives having the following diketopiperazinylmethyl groups in place of the (4-ethyl-2,3-dioxo-1-piperazinecarboxamido) methyl group in **1a**: (4-ethyl-2,3-dioxopiperazin-1-yl) methyl (K-852 (**6**)), (cyclo-L-prolylglycinyl) methyl (K-854 (**7**))^29–31^ and (4-methyl-2,5-dioxopiperazin-1-yl) methyl (K-856 (**8**)). Additionally, OP-857 (**9**) and OP-858 (**10**) with a 3-oxopiperazinylmethyl group and OP-859 (**11**) with the 4-ethylpiperazinylmethyl group were prepared to examine the SAR of these compounds. The K-560 analogues **3**, **4**, and **5** were prepared starting from *tert-*butyl 2-amino-4-bromophenylcarbamate, as shown in Fig. 1. This starting material was subjected to Suzuki cross-coupling with phenylboronic acid, 3-furanboronic acid, and 2-furanboronic acid in the presence of *tri-o-*tolylphosphine, K_2_CO_3_, and tetrakis(triphenyl phosphine) palladium, giving biaryls **3a**, **4a**, and **5a**, respectively. These aryls were condensed with *p-*(chloromethyl) benzoyl chloride in the presence of triethylamine (Et_3_N), yielding the chlorides **3b**, **4b**, and **5b**, respectively. The chlorides, after conversion to the 4-((1, 3-dioxoisoindolin-2-yl) methyl) benzamide derivatives **3c**, **4c**, and **5c**, were reduced with hydrazine to the 4-(aminomethyl) benzamide compounds **3d**, **4d**, and **5d**, respectively. These amines were treated with 4-ethyl-2,3-dioxo-1-piperazinecarbonyl chloride in the presence of Et_3_N and afforded the condensation products **3e**, **4e**, and **5e**, which were, in turn, Boc-deblocked with trifluoroacetic acid (TFA) to yield K-562 (**3**), K-563 (**4**), and K-564 (**5**), respectively. On the other hand, the K-560 derivatives **6**, **7**, **8**, **9**, **10** and **11** were synthesized starting from *tert-*butyl 2-(4-(chloromethyl) benzamido)-4-(thiophen-2-yl) phenylcarbamate, as shown in Fig. 2. This starting material was reacted with 4-ethyl-2,3-dioxo-1-piperazine, cyclo-L-prolylglycine, 1-methylpiperazine-2,5-dione, 1-methyl-piperazin-2-one, *tert-*butyl 3-oxopiperazine-1-carboxylate and 1-ethylpiperazine in the presence of sodium hydride (NaH) to yield the condensation products **6a**, **7a**, **8a**, **9a**, **10a** and **11a**, which were, in turn, Boc-deblocked with TFA to give the desired K-852 (**6**), K-854 (**7**), K-856 (**8**), OP-857 (**9**), OP-858 (**10**) and OP-859 (**11**), respectively.

### HDAC1, 2, 3, 6 and 8 inhibition of HDAC inhibitors

Table 1 shows the inhibitory activities (IC_50_s) of the synthesized compounds together with **1a** and TSA against HDAC1, 2, 3, 8 (Class I) and 6 (Class II), which were measured according to the protocol of Enzo Life Sciences. All the compounds except for TSA inhibited preferentially HDAC1 and HDAC2 over HDAC3, 6 and 8. However, their IC_50_ values of HDAC2 inhibition were (10–26-fold) greater than those of HDAC1 inhibition.

**Table 1:** Inhibitory activities of HDAC inhibitors against activation of HDAC1, 2, 3, 8 and 6.

Compound	IC_50_ (μM)				
	Class I				Class II
	HDAC1	HDAC2	HDAC3	HDAC8	HDAC6
K-560 (**1a**)	0.077	0.77	>25	>25	>25
K-562 (**3**)	0.063	0.81	>25	>25	>25
K-563 (**4**)	0.061	0.73	>25	>25	>25
K-564 (**5**)	0.066	1.44	>25	>25	>25
K-852 (**6**)	0.068	0.98	>25	>25	>25
K-854 (**7**)	0.063	1.22	>25	>25	>25
K-856 (**8**)	0.086	1.11	>25	>25	>25
TSA	0.020	0.018	0.163	—	—
OP-857 (**9**)	0.131	1.58	>25	>25	>25
OP-858 (**10**)	0.186	2.05	>25	>25	>25
OP-859 (**11**)	0.092	2.43	>25	>25	>25
TSA	0.032	0.024	0.451	1.18	0.204

Following two groups of experiments were conducted independently: (one group) HDAC1-3 inhibition of **1a**, **3**, **4**, **5**, **6**, **7**, **8**; (the other group) HDAC6, 8 inhibition of **1a**, **3**, **4**, **5**, **6**, **7**, **8**, **9**, **10**, **11** and HDAC1-3 inhibition of **9**, **10**, **11**. TSA was used as a positive control.

### Ameliorating effects of HDAC inhibitors on cell damage after oxygen-glucose deprivation

**Figure 3:**
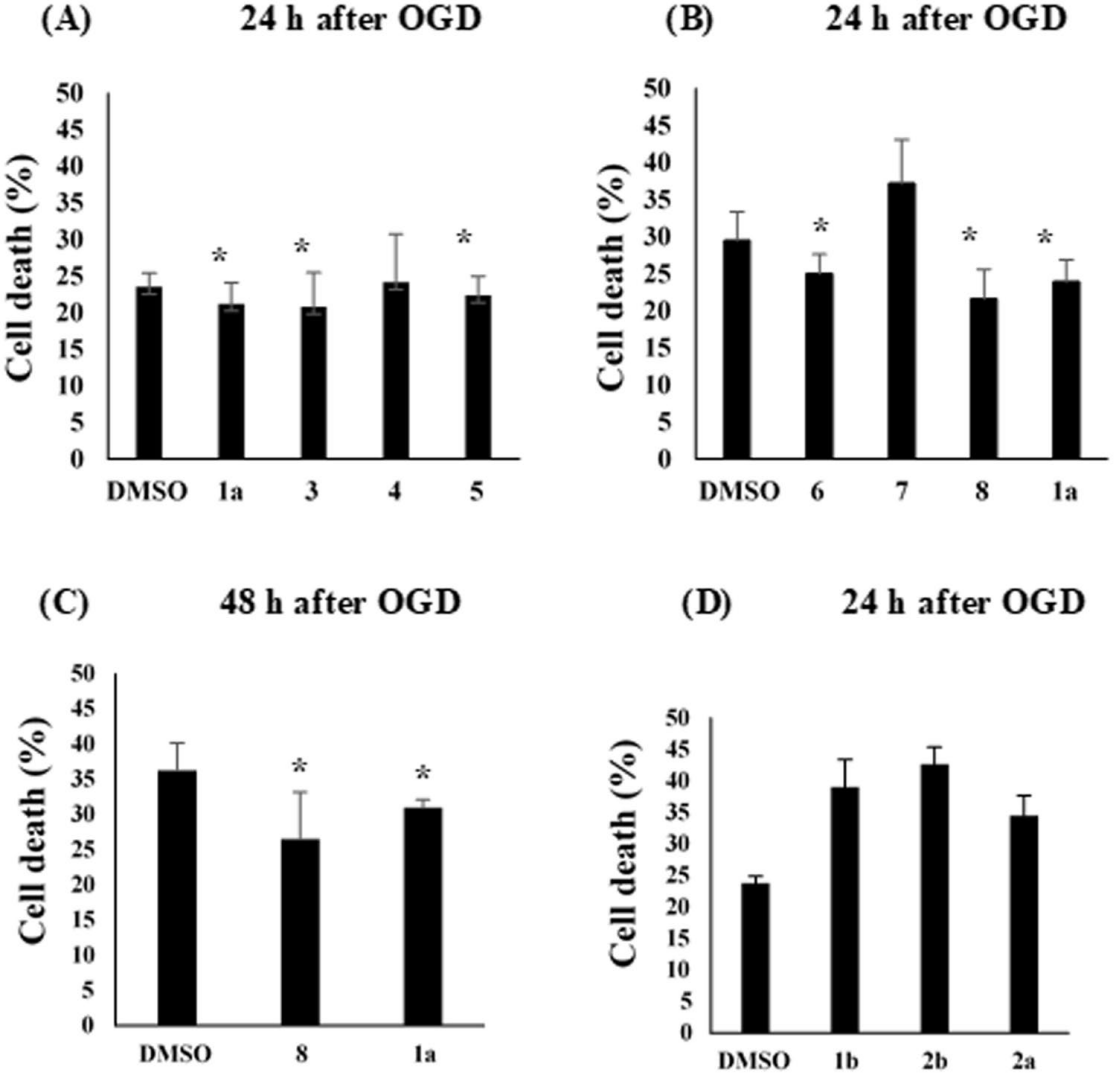
Evaluation of Percentages of Cell Death in Primary Cultures of Rat Cortical Neurons Exposed to HDAC Inhibitors. Cell death (%) in primary cultures of rat cortical neurons was determined by measuring LDH release 24 h or 48 h after OGD. Cultures were incubated with (**A**) **1a**, **3**, **4** or **5** each at 1 μM; (**B**) **6**, **7**, **8** or **1a** each at 1 μM; (**C**) **8** or **1a** each at 1 μM; (**D**) **1b**, **2a** or **2b** each at 1 μM, under the conditions noted in the section of Materials and Methods. Each value is the mean ± standard error mean of triplicate measurements. The asterisk denotes a significant difference (*p < 0.05) *versus* the control (0.1% DMSO).

**Figure 4:**
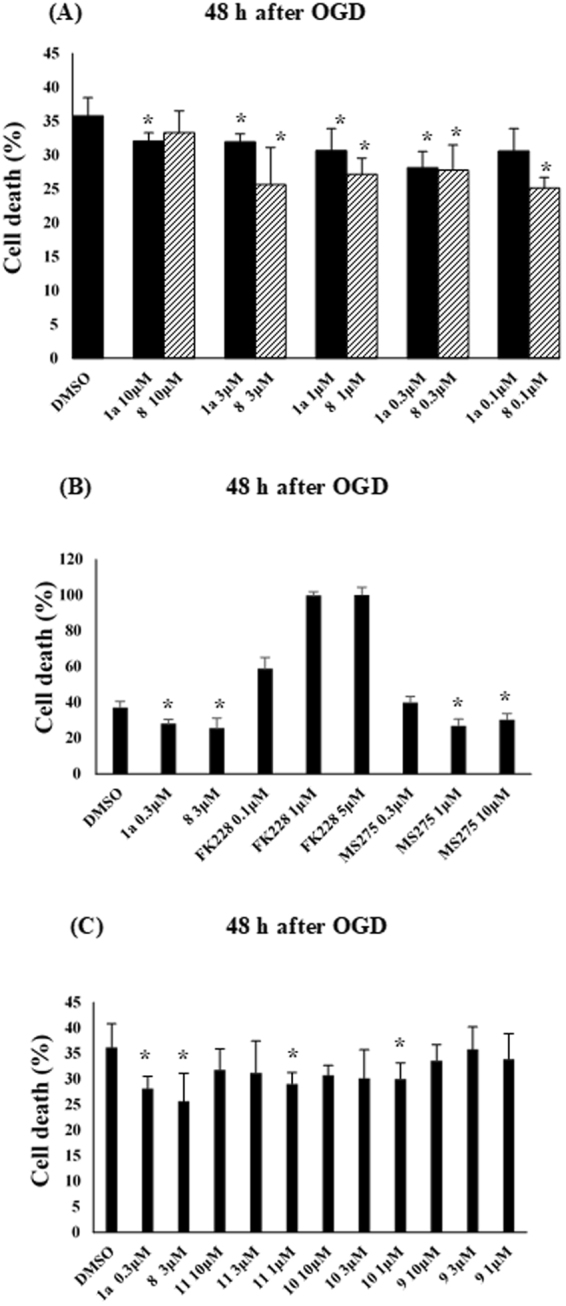
Dose-Dependent Evaluation of Percentages of Cell Death in Primary Cultures of Rat Cortical Neurons Exposed to HDAC Inhibitors. Cell death (%) in primary cultures of rat cortical neurons was determined by measuring LDH release 48 h after OGD. Cultures were incubated with (**A**) **8** or **1a** each at 0.1, 0.3, 1, 3 or 10 μM; (**B**) FK228 (at 0.1, 1 or 10 μM), MS-275 (at 0.3, 1, 10 μM), **1a** (at 0.3 μM) and **8** (at 3 μM); (**C**) **9**, **10**, **11** (each at 1, 3 or 10 μM), **1a** (at 0.3 μM) and **8** (at 3 μM), under the conditions noted in the section of Materials and Methods. Each value is the mean ± standard error mean of triplicate measurements. The asterisk denotes a significant difference (*p < 0.05) *versus* the control (0.1% DMSO).

Primary rat cortical neurons prepared as indicated in the section of Materials and Methods were pre-incubated with a tested compound for 1 h and then subjected to oxygen-glucose deprivation (OGD) injury for 3 h. Neuronal cell death was assessed by performing a lactate dehydrogenase (LDH) assay 24 or 48 h after ischemia. Figure 3A shows the percentage of neuronal cell death resulting from incubation with K-560 (**1a**), K-562 (**3**), K-563 (**4**), or K-564 (**5**) at 1 μM, and Fig. 3B depicts the percentage of cell death resulting from incubation with K-852 (**6**), K-854 (**7**), K-856 (**8**), or K-560 (**1a**) at 1 μM. The results obtained revealed that **3**, **5**, **6**, and **8**, as well as **1a**, exerted protective effects (LDH assay in 24 h) against OGD-induced damage. Since K-856 (**8**) seemingly exerted the most promising protective effects (though with no significant difference between them) (Fig. 3B), its protective activity was compared with that of **1a** by performing the LDH assay 48 h after ischemia (Fig. 3C). It is noteworthy that both K-560 (**1a**) and K-856 (**8**) maintained a lower percentage of neuronal cell death than the control (0.1%DMSO) even in 48 h. Figure 3D shows the percentage death of neuronal cells which were incubated with the 2-nitro form^4^ (**1b**) of K-560 (**1a**), K-561 (**2a**), or the 2-nitro form^4^ (**2b**) of **2a**. None of these compounds exerted protective effects and, instead, they decreased cell viability compared with the control (by the LDH assay 24 h after ischemia). In order to monitor the changes in the protective effects of **1a** and **8** with the concentrations, cortical neurons were incubated with them at ranging from 0.1 to 10 μM before OGD-induced injury, respectively, (Fig. 4A). It was substantiated that, although dose-dependency was not clearly observed, **1a** and **8** reduced the percentages of cell death at 10–0.3 μM and at 3–0.1 μM (with significant differences *vs*. control), respectively, and that **8** was more effective than **1a** at the lowest concentration (0.1 μM). Furthermore, it is suggested from the comparative experiment (Fig. 4B) with the clinically-used HDAC inhibitors, FK228 (HDAC1,2 selective inhibitor), MS-275 (HDAC Class I inhibitor), as well as **1a** and **8**, that **1a** and **8** are more potent than FK228, since FK228 was more toxic even at 0.1 μM than the control, whereas they have nearly the same level of protective activity as MS-275. Figure 4C indicates that none of the 4-methyl-substituted 3-oxopiperazine form: OP-857 (**9**), the 4-unsubstituted 3-oxopiperazine-form: OP-858 (**10**) and the 4-ethylpiperazine-form: OP-859 (**11**) was effective with significant difference from the control, though compound **10** looked more effective than **9** and **11**.

### Assessment of toxicity degree of HDAC inhibitors for neuronal SH-SY5Y cells by monitoring cell population of Sub-G_0_/G_1_ phase

**Figure 5:**
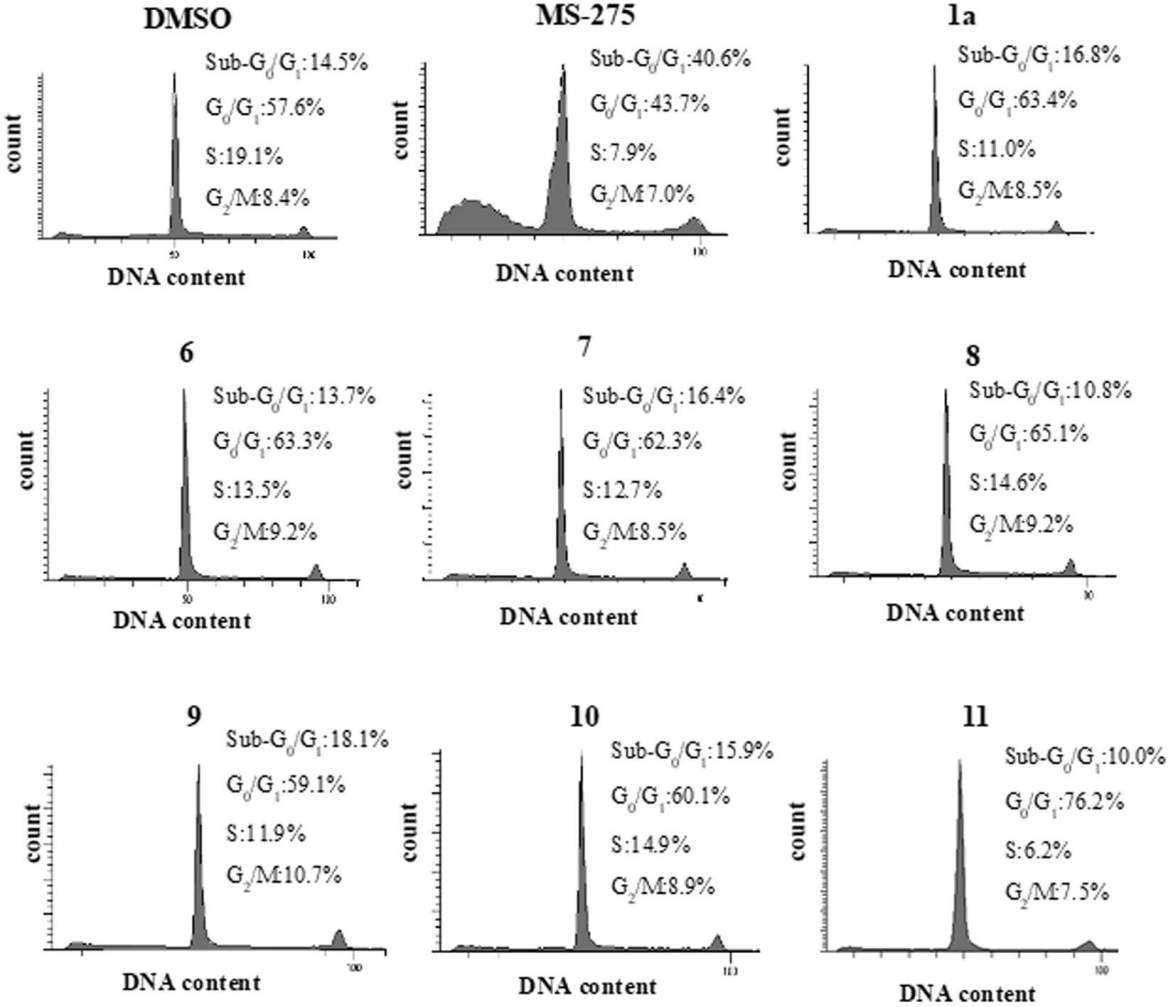
Assessment of Toxicity Degree of HDAC inhibitors for SH-SY5Y Cells by Flow Cytometry. SH-SY5Y cells were treated with **1a**, **3**, **4**, **5**, **6**, **8**, **9**, **10** or **11** for 48 h as indicated in the section of Material and Methods, and their DNA contents were analyzed using flow cytometry. The percentages of the population in the Sub-G_0_/G_1_, G_0_/G_1_, S and G_2_/M phase are indicated. 0.1%DMSO was used as a control. The experiment was repeated three times and representative histograms are shown.

The toxicity of the HDAC1, 2 inhibitors to SH-SY5Y cells were estimated by monitoring the percentage of population of Sub-G_0_/G_1_ phase in the SH-SY5Y cells. Thus, the cells, after treatment with **1a**, **3**, **4**, **5**, **6**, **8**, **9**, **10** or **11** for 48 h, were subjected to flow cytometric analyses. As a positive control, MS-275 was used. Figure 5 shows the percentages of the population in the Sub-G_0_/G_1_, G_0_/G_1_, S and G_2_/M phase for each compound. Among them, MS-275 showed the highest percent (40.6%) of cell distribution of Sub-G_0_/G_1_ phase, whereas the other compounds exhibited more or less the same level of Sub-G_0_/G_1_ distribution (10.0–18.1%) as that of the control (14.5%). These results suggest that **8** has a low toxicity to SH-SY5Y cells and is much less toxic than MS-275.

## Discussion

Three hydroxamate-type pan-HDAC inhibitors have been approved by the US Food and Drug Administration (FDA) to date for the treatment of the following cancers: SAHA for the advanced forms of cutaneous T-cell lymphoma (CTCL)^5,6,32^; Belinostat (Beleodaq) for refractory peripheral T-cell lymphoma (PTCL)^33^; Panobinostat (Farydak) for the combinatorial treatment of multiple myeloma^34^. Furthermore, the cyclic depsipeptide FK228 (Romidepsin) was licensed by the US FDA for the treatment of CTCL^5,6,32^, and the 2-aminobenzamide-type HDAC inhibitor MS-275 (Entinostat), for breakthrough therapy in the treatment of advanced breast cancers^35^. On the other hand, HDAC inhibitors have emerged as an attractive therapeutic candidate for neurodegeneration in the last decade^36,37^ because therapeutic options for neuroprotective therapies in subacute or chronic ischemic stroke currently remain very limited. It is anticipated that HDAC inhibitors may act against chronic neurodegeneration without tumorigenesis because they have been developed as treatment drugs for various cancers. Endovascular therapy after intravenous t-PA has been reported to lead to better functional outcomes^38–41^. Although pan-HDAC inhibitors such as SAHA and TSA were previously shown to prevent ischemic cell damage and improve functional recovery, they may cause side effects due to the non-selectivity of their HDAC isoforms. Since HDAC1, 2, and 3 are strongly expressed in the central nervous system, a promising strategy may be to develop selective inhibitors of these enzymes in order to assess their effectiveness as a new therapeutic agent for ischemic stroke.

**Figure 6:**
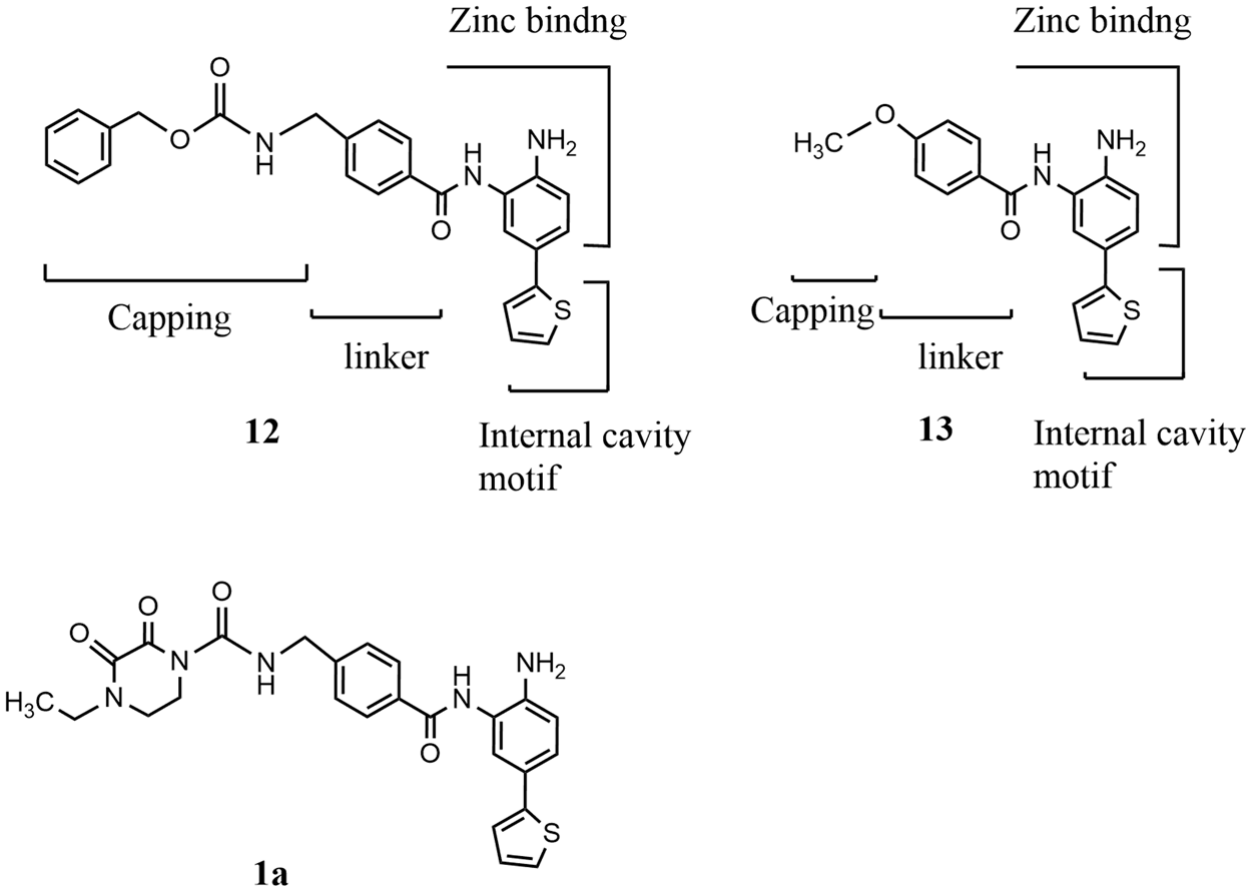
Structural Regions of 5-Thienyl-Substituted 2-Aminobenzamide type HDAC Inhibitors.

We were encouraged by the result showing that the HDAC1, 2-selective inhibitor K-560 (**1a**) exerted neuroprotective effects^23–25^. Therefore, we attempted to synthesize K-560-related compounds in order to assess their inhibitory activities against HDAC1, 2, 3, 8 (Class I) and 6 (Class II) and ameliorating effects on *in vitro* cerebral ischemia and also to examine their SAR. The results obtained revealed that all the compounds tested preferentially inhibited HDAC1 and 2 though with a 10–26-fold selectivity for HDAC1 over HDAC2. According to the SAR study between 5-thienyl-substituted 2-aminobenzamide HDAC1,2 inhibitors, even truncation (exemplified by **13**) of the capping group and linker domain of compound **12** maintained a high selectivity for HDAC1 and HDAC2 versus HDAC3 and HDAC8, but reduced the preference for HDAC1 versus HDAC2^42^ (Fig. 6). It is well known that the cap region of HDAC is predominantly responsible for selectivity^43^. Based on these findings, it is seems reasonable that our HDAC1,2 inhibitors, typified by **1a**, having almost the same size of capping and linker domains as those of **12**, showed the preferential inhibition for HDAC1 over HDAC2.

The OGD experiments indicated that the synthesized HDAC1, 2 inhibitors and K-560 (**1a**) exerted neuronal cell protection except for K-563 (**4**), K-854 (**7**), OP-857 (**9**), OP-858 (**10**) and OP-859 (**11**) (Figs 3A,B,C and 4C). It was also revealed that **8** and **1a** had the promising neuroprotective activity, but the effect of **8** was higher than or at least equal to **1a**. This finding was supported from the comparative OGD experiment with various concentrations of **8** and **1a**, *i.e*., it showed that **8** was still effective at the lowest concentration (0.1 μM) where **1a** was not effective (Fig. 4A). Another comparative experiment using the clinically used medicines FK228 (HDAC1, 2 inhibitor) and MS-275 (HDAC Class I inhibitor) suggested that both **8** and **1a** have a higher neuroprotective activity than FK228, whereas they possess nearly the same activity as MS-275 (Fig. 4B). Since, however, MS-275 showed much higher percent (40.6%) of distribution of Sub-G_0_/G_1_ phase cell (corresponding to apoptotic cell) than those (10.0–18.1%) for other compounds in the flow cytometric analyses of neuronal SH-SY5Y cells (Fig. 5), compound **8** is expected to be less toxic than MS-275 to neuronal cells.

Because the cyclo-L-prolylglycinylmethyl group reportedly exhibited both nootropic and anxiolytic activities^29^, it was unexpected that compound **7**, which possesses this group, was not effective. We speculate that a bicyclic diketopiperazine ring such as that in **7** may be less suitable than a monocyclic diketopiperazine ring for interactions with biological molecules due to its bulkiness. Furthermore, since the HDAC1 and 2 inhibition of **1a** and **3**–**11** was comparable (Table 1), it would be inappropriate that only their enzyme inhibition activities have responsibility for the neuroprotective effects. Instead, it is reasonable to assume that the diketopiperazine moieties (capping group) in these compounds are at least partially involved in their neuronal protection, in view of the reports that diketopiperazines themselves had neuroprotective activities^26–28^. This assumption was supported by the finding that neither the monoketopiperazine-form **9** and **10** nor the piperazine-form **11** showed the neuroprotective effect as much as the diketopiperazine-form **8** and **1a** (Fig. 4C).

The 2-nitro derivatives **1b** and **2b** as well as **2a**, having the (4-methylpiperazine-1-carboxamido) methyl moiety, deteriorated cell viability from that with the control (Fig. 3D). The 2-amino group in the 2-amino-5-aryl-benzamide type HDAC inhibitor is known to play a role in chelation with zinc ion at the active site of the HDAC enzyme together with the 2-aminobenzamide-carbonyl, leading to its inhibition. Taken together, the presence of a monocyclic 2,3- or 2,5-diketopiperazine group as well as the HDAC1, 2 inhibition by the 2-aminobenzamide moiety appears to be crucial for the cell survival activity of K-560-related compounds against ischemic damage.

HDAC1 is a key molecular player between neuronal survival and death^44,45^. HDAC2 abolishes neurodegeneration-associated memory impairments *via* epigenetic blockade^46,47^, and mitigates remote fear memories^48^. In ischemic stroke, the inhibition of both or either HDAC1 and HDAC2 reduce ischemic damage. In neurological disorders, it is currently unclear which isozyme of HDAC enzymes needs to be inhibited because the type of HDAC being activated varies depending on the distinct pathophysiology, associated cell types, or the degree and severity of tissue damage. Further studies are underway to assess the validity of K-560-related compounds for CNS therapeutics against neurodegenerative diseases and stroke.

## Materials and Methods

The experimental protocol was approved by the Committee of Osaka University Graduate School of Medicine, Kansai University, and Osaka University of Pharmaceutical Sciences.

### Synthesis

The syntheses and physicochemical properties of HDAC inhibitors are provided as Supplementary information available with this article online.

### Biology

#### Assessment of HDAC inhibition activities

The inhibitory activities of compounds against HDAC1, 2, 3, 8 (Class I) and 6 (Class II) were measured utilizing the Fluorometric Drug Discovery Assay Kit BML-AK511 (HDAC1), BML-AK 512 (HDAC2), and BML-AK 531 (HDAC3), BML-SE145-0100 (HDAC8) and BML-SE508-0050 (HDAC6) (supplied from Enzo Life Sciences). The following two groups of experiments were conducted independently: (one group) HDAC1-3 inhibition of **1a**, **3**, **4**, **5**, **6**, **7** and **8**; (the other group) HDAC6, 8 inhibition of **1a**, **3**, **4**, **5**, **6**, **7**, **8**, **9**, **10** and **11** and HDAC1-3 inhibition of **9**, **10**, **11**. TSA was used as a positive control in each group. Each assay was independently repeated two times with duplicate measurements, and a similar value was obtained for each compound.

#### Cell culturing

Human neuroblastoma cell line SH-SY5Y (ATCC CRL-2266) was cultured in Dulbecco’s Modified Eagle’s Medium (Sigma-Aldrich) supplemented with 10% fetal bovine serum (Gibco) at 37 °C in a 95% air and 5% CO_2_ humidified incubator. Cells were routinely subcultured when confluent.

#### Animals

Wistar rats (Charles River) were used in this study. The experimental protocol was approved by the institutional animal care and use committee of Osaka University Graduate School of Medicine. Animals were kept four per cage under a 12 h light/dark cycle and standard housing conditions with ad libitum access to food and water before and after all procedures. Animal care was provided according to the Osaka University Medical School Guideline for the Care and Use of Laboratory Animals. Animal surgeries and experimental procedure were approved by the Osaka University Medical School Animal Care and Use Committee. All experiments were conducted according to the National Research Council’s guidelines.

#### Primary cortical cultures

Primary cultures of rat cortical neurons were obtained as described previously^49,50^. Briefly, neuronal cultures were prepared from the cortex of embryonic day 16 (E16) rat embryos. Cells were dissociated with papain (papain dissociation system; Worthington) and plated onto 12-well plates, 4-well plates, and 60-mm dishes (Falcon, Becton Dickinson), or 4-chamber glass slides (Falcon) coated with polyethylenimine. Cells at a final concentration of 7.0 × 10^5^ cells/mL were cultured in high-glucose DMEM (Sigma) containing 10% fetal calf serum (FCS; Invitrogen), 100 IU/mL penicillin, and 100 μg/mL streptomycin sulfate. Twenty-four hours after seeding, the medium was changed to Neurobasal medium (Invitrogen) supplemented with B-27 (Invitrogen). Cells were cultured at 37 °C in a humidified atmosphere of 95% air and 5% CO_2_ and used after 10–11 days *in vitro* when most cells showed a neuronal phenotype.

#### Oxygen-glucose deprivation (OGD)

OGD was performed by placing cultures in a 37 °C incubator housed in an anaerobic chamber as previously described^49,50^. Cultures were washed with phosphate-buffered saline and incubated with glucose-free Eagle’s balanced salt solution (Biological Industries). Cultures were subjected to an anaerobic environment of 95% and N_2_/5% CO_2_, producing an O_2_ partial pressure of 10–15 Torr, as measured with an oxygen microelectrode at 3 h. OGD was terminated by bringing the cultures back to the original medium and placing them in a normoxic chamber.

#### Cell viability Assays

Before OGD, cortical neurons were treated with the following chemicals each at 1 μM for 1 h: K-560 (**1a**), K-562 (**3**), K-563 (**4**) and K564 (**5**) (Fig. 3A); K-560 (**1a)**, K-852 (**6**), K-854 (**7**) and K-856 (**8**) (Fig. 3B); **1a** and **8** (Fig. 3C); **1b**, **2b** and **2a** (Fig. 3D). Furthermore, cortical neurons were treated for 1 h with **1a** and **8** each at 0.1, 0.3, 1, 3 or 10 μM (Fig. 4A), with FK228 (Selleck Chemicals) (at 0.1, 1 or 10 μM), MS-275 (at 0.3, 1, 10 μM), **1a** (at 0.3 μM) and **8** (at 3 μM) (Fig. 4B) and with OP-857 (**9**), OP-858 (**10**), OP-859 (**11**) (each at 1, 3 or 10 μM), **1a** (at 0.3 μM) and **8** (at 3 μM) (Fig. 4C). As the control, 0.1%DMSO was used in each experiment. Neuronal injury was measured 24 h or 48 h after OGD by measuring LDH activity using a cytotoxicity detection kit (Roche Applied Science, Mannheim, Germany). Collected culture medium was centrifuged at 300 × *g* for 5 min before assaying according to the manufacturer’s instructions. In a sister culture, 100% cell death was induced with 2 mmol/L NMDA. The relative assessments of neuronal injury were normalized by comparisons with 100% cell death.

#### Analysis of DNA histogram by flow cytometry

SH-SY5Y cells were plated onto 60-mm diameter dishes (1.0 × 10^6^/dish). After incubation for 24 h, the cells were washed twice with the serum-free medium (1 mL) and suspended with the serum-free medium (5 mL) for 24 h. After removal of the medium, the cells were washed twice with the serum-free medium (1 mL) and incubated in the serum-free medium (5 mL) with a test compound (10 μM) for another 48 h. The adherent cells were treated with 0.25% trypsin (Invitrogen) and combined with the floating cells. All the cells were treated with a Cycle Test Plus DNA reagent Kit (Catalog No. 340242, Becton Dickinson). DNA content was measured with a FACSTMCant II. (Becton Dickinson).

#### Statistical analysis

Data are expressed as the mean ± standard error mean. Statistical analyses were performed using a one-way analysis of variance (SPSS). A *p*-value of less than 0.05 denotes a significant difference.

## Electronic supplementary material

Supplementary information
