# Supplementary material for: New 5-Aryl-Substituted 2-Aminobenzamide-Type HDAC Inhibitors with a Diketopiperazine Group and Their Ameliorating Effects on Ischemia-Induced Neuronal Cell Death
Source: Sci Rep. 2018 Jan 23;8:1400. doi: 10.1038/s41598-018-19664-9 (PMC5780423; doi:10.1038/s41598-018-19664-9)
Supplement: Supplementary file 1 — Supplementary information [file 41598_2018_19664_MOESM1_ESM.doc]

**New 5-Aryl-Substituted 2-Aminobenzamide-Type HDAC Inhibitors with a Diketopiperazine Group and Their Ameliorating Effects on Ischemia-Induced Neuronal Cell Death.**

Yoshiyuki Hirata^1,2^, Tsutomu Sasaki*^3^, Hideaki Kanki^3^, Chi-Jing Choong ^3^, Kumiko Nishiyama^3^, Genki Kubo^1^, Ayana Hotei^1^, Masahiko Taniguchi^2^, Hideki Mochizuki^3^, Shinichi Uesato*^1,2^

^1^Department of Life Science and Biotechnology, Faculty of Chemistry, Materials and Bioengineering, Kansai University, Suita, Osaka 564-8680, Japan. ^2^Osaka University of Pharmaceutical Sciences, 4-20-1 Nasahara, Takatsuki, Osaka 569-1094, Japan. ^3^Department of Neurology, Graduate School of Medicine, Osaka University, Yamadaoka 2-2, Suita, Osaka 565-0871, Japan.

**Supplementary information**

**Synthesis**

**General methods.** Melting points were assessed on a Yanagimoto MP-500P micromelting point apparatus (Yanako) and were uncorrected. High-resolution (HR)-ESI-MS spectra were recorded on LCMS-IT-TOF (Shimadzu). ^1^H NMR spectra were recorded on JEOL EX-400 (399.65 MHz) and Agilent 400-MR DD2 (399.85 MHz) in CDCl_3_, unless otherwise noted, as a solvent and tetramethylsilane as an internal standard. Purity of samples was determined using a Nexera HPLC-system (Shimadzu). Compounds were analyzed using inertsil ODS-3 (2.1 mm × 100 mm, 3 μm) with UV detection (280 nm) under the following conditions. Compounds **3**, **4**, **5**, **6**, **7** and **8**: mobile phase, 30% MeCN-H_2_O (0 - 6 min), 80% MeCN-H_2_O (6 - 8 min), 30% MeCN-H_2_O (8 - 10 min); flow rate, 0.25 mL/min. Compound **9**: mobile phase, 50% MeCN-H_2_O; flow rate, 0.2 mL/min. **10**: mobile phase, 60% MeCN-H_2_O; flow rate, 0.2 mL/min. **11**: mobile phase, 30% MeCN-H_2_O; flow rate, 0.2 mL/min. Analytical TLC was performed using a Silica gel 60 F254 (Merck, 0.25 mm) glass plate. Silica gel chromatography was performed using the Automated Flash Chromatography System: W-Prep 2XY-10VW (Yamazen) and Biotage ZIPTM (Biotage) as a disposable cartridge. Preparative TLC (PLC) were performed using Silica gel 60 F254 (Merck, 1 mm) glass plates. All solvents were dried over Na_2_SO_4_ and evaporated *in vacuo*. MS-275 was prepared by our group according to the reported procedure.^1^ HPLC purity: >99%.

1. Suzuki, T. et al. Synthesis and histone deacetylase inhibitory activity of new benzamide derivatives. *J. Med. Chem.* 42, 3001-3003 (1999).

***tert-*Butyl 3-aminobiphenyl-4-ylcarbamate (3a).** A solution of K_2_CO_3_ (5.55 g, 40.2 mmol) (18 mL) and tetrakis (triphenylphosphine) palladium (0.9 g, 0.78 mmol) in H_2_O (18 mL) were added to a solution of *tert-*butyl 2-amino-4-bromophenylcarbamate (3.00 g, 10.5 mmol), phenylboronic acid (2.15 g, 17.6 mmol), and *tri-o-*tolylphosphine (1.35 g, 4.4 mmol) in DME (30 mL). After stirring at 90°C for 18 h, the reaction suspension was mixed with CHCl_3_, washed with H_2_O, dried, and concentrated. The resultant residue was chromatographed over silica gel with ethyl acetate (EtOAc)/n-hexane 3:7 as a developing solvent, yielding **3a** (2.10 g, 7.4 mmol, 70%) as pale yellowish crystals. mp: 153-154°C. ^1^HNMR δ: 1.48 (9H, s, CH_3_ ×3), 3.81 (2H, brs, NH_2_), 6.28 (1H, brs, NH), 6.98-7.23 (2H, m, Ar-H_2_), 7.29-7.42 (4H, m, Ar-H_4_), 7.52-7.54 (2H, m, Ar-H_2_). HR-ESI-MS (m/z): 285.1601 (M+H)^+^ calcd for C_17_H_21_BN_2_O_2_ 285.1603.

***tert-*Butyl 3-(4-(chloromethyl)benzamido)biphenyl-4-ylcarbamate (3b).** *p-*(Chloromethyl)benzoyl chloride (0.80 g, 4.2 mmol) was added to a solution of **3a** (1.0 g, 3.52 mmol) in THF (14 mL) containing Et_3_N (0.57 mL) under ice-cooling. After stirring at room temperature (RT) for 4 h, the mixture was dissolved in CHCl_3_. The CHCl_3_ solution was successively washed with satd. NaHCO_3_ and brine, dried, and concentrated. The resultant product was chromatographed over silica gel with EtOAc/n-hexane 1:2 to afford **3b** (1.1 g, 2.52 mmol, 71%) as colorless crystals. mp: 140.2-141.3°C. ^1^HNMR δ: 1.45 (9H, s, CH_3_ ×3), 4.61 (2H, s, CH_2_), 6.95 (1H, brs, NH), 7.26-7.61 (9H, m, Ar-H_9_), 7.96-7.98 (3H, m, Ar-H_3_), 9.37 (1H, brs, NH). HR-ESI-MS (m/z): 435.1480 (M-H)^-^ calcd for C_25_H_24_ClN_2_O_3_ 435.1476.

***tert-*Butyl 3-(4-((1, 3-dioxoisoindolin-2-yl)methyl)benzamido)biphenyl-4-ylcarbamate (3c).** Potassium phthalimide (234 mg, 1.3 mmol) and KI (38 mg, 0.23 mmol) were added to a solution of **3b** (500 mg, 1.15 mmol) in DMF (5 mL), and the solution was stirred at 50°C overnight. The evaporation of DMF gave a residue, which was dissolved in EtOAc. The EtOAc solution was successively washed with brine, dried, and concentrated. The resultant product was purified by silica gel chromatography with EtOAc/n-hexane 1:2 as an eluent, giving **3c** (595 mg, 1.09 mmol, 95%) as pale yellowish crystals. mp: 104-106°C. ^1^HNMR δ: 1.46 (9H, s, CH_3_ ×3), 4.90 (2H, s, CH_2_), 6.85 (1H, brs, NH), 7.26-7.39 (5H, m, Ar-H_5_), 7.49-7.56 (4H, m, Ar-H_4_), 7.72-7.75 (2H, m, Ar-H_2_), 7.86-7.88 (2H, m, Ar-H_2_), 7.92-7.97 (3H, m, Ar-H_3_), 9.18 (1H, brs, NH). HR-ESI-MS (m/z): 546.2037 (M-H)^-^ calcd for C_33_H_28_N_3_O_5_ 546.2029.

***tert-*Butyl 3-(4-(aminomethyl)benzamido)biphenyl-4-ylcarbamate (3d).** Hydrazine monohydrate (0.1 mL, 2.7 mmol) was added to a suspension of **3c** (500 mg, 0.91 mmol) in EtOH (6 mL), and the mixture was stirred under reflux for 3 h. The evaporation of EtOH gave a residue, which was dissolved in CHCl_3_. The CHCl_3_ solution was successively washed with satd. NaHCO_3_ and brine, dried, and concentrated. The resultant product was purified by silica gel chromatography with CHCl_3_/MeOH 9:1 as an eluent, affording **3d** (171 mg, 0.41 mmol, 45%) as pale yellowish crystals. mp: 106-108°C. ^1^HNMR δ: 1.46 (9H, s, CH_3_ ×3), 3.94 (2H, s, CH_2_), 6.91-8.06 (13H, m, Ar-H_12_, NH), 9.31 (1H, brs, NH). HR-ESI-MS (m/z): 418.2120 (M+H)^+^ calcd for C_25_H_28_N_3_O_3_ 418.2130.

***tert-*Butyl 3-(4-((4-ethyl-2, 3-dioxopiperazine-1-carboxamido)methyl)benzamido)biphenyl-4-ylcarbamate (3e).** 4-Ethyl-2,3-dioxo-1-piperazinecarbonyl chloride (88 mg, 0.43 mmol) was added to a solution of **3d (**150 mg, 0.36 mmol) in CH_2_Cl_2_ (2 mL) containing Et_3_N (0.6 mL). After stirring at RT overnight, the reaction mixture was concentrated. The residue was dissolved in CHCl_3_ and successively washed with satd. NaHCO_3_ and brine, dried, and concentrated. The resultant product was purified by silica gel chromatography with CHCl_3_/MeOH 98:2 containing Et_3_N 0.35%, yielding **3e** (113 mg, 0.19 mmol, 53%) as pale yellowish crystals.

mp: 217-218°C. ^1^HNMR δ: 1.21 (3H, t, *J =* 7.0 Hz, CH_3_), 1.51 (9H, s, CH_3_ ×3), 3.53-3.67 (4H, m, CH_2_ ×2), 4.08-4.10 (2H, m, CH_2_), 4.56-4.58 (2H, m, CH_2_), 7.07-7.97 (13H, m, Ar-H_12_, NH), 9.31 (1H, brs, NH), 9.39 (1H, brs, NH). HR-ESI-MS (m/z): 584.2511 (M-H)^-^ calcd for C_32_H_34_N_5_O_6_ 584.2509.

***N-*(4-(4-Aminobiphenyl-3-ylcarbamoyl)benzyl)-4-ethyl-2, 3-dioxopiperazine-1-carboxamide (3) (K-562).** Compound **3e** (100 mg, 0.21 mmol) was dissolved in a mixture of TFA and CH_2_Cl_2_ (1:1) (2 mL). After stirring at RT for 1 h, the solution was successively adjusted to pH 9 with satd. NaHCO_3_, stirred for 1 h, washed with brine, dried, and concentrated. The solid product was recrystallized from EtOH to give **3** (96 mg, 0.20 mmol, 95%) as pale yellowish crystals. mp: 119-121°C. ^1^HNMR δ: 1.13-1.16 (3H, m, CH_3_), 3.43-3.50 (4H, m, CH_2_ ×2), 3.97-4.00 (2H, m, CH_2_), 4.03 (2H, brs, NH_2_), 4.50 (2H, d, *J =* 6.0 Hz, CH_2_), 6.83 (1H, d, *J =* 8.0 Hz, Ar-H), 7.23-7.54 (9H, m, Ar-H_9_), 7.86 (2H, d, *J =* 8.0 Hz, Ar-H_2_), 8.45 (1H, brs, NH), 9.35 (1H, brs, NH). HR-ESI-MS (m/z): 486.2137 (M+H)^+^ calcd for C_27_H_28_N_5_O_4_ 486.2141. HPLC purity: >90% (Rt: 7.3 min)

***tert-*Butyl 2-amino-4-(furan-3-yl)phenylcarbamate (4a).** *tert-*Butyl 2-amino-4-bromophenylcarbamate (3.0 0 g, 10.4 mmol), 3-furanboronic acid (1.51g, 13.5 mmol), and *tri-o-*tolylphosphine (1.35 g, 4.4 mmol) were reacted with K_2_CO_3_ (5.55 g, 40.2 mmol) and tetrakis(triphenyl phosphine)palladium (0.90 g, 0.78 mmol) in the same manner as described for the preparation of **3a**, giving **4a** (2.40 g, 8.7 mmol, 84%) as pale yellowish crystals. mp: 140-141°C. ^1^HNMR δ: 1.52 (9H, s, CH_3_ ×3), 3.78 (2H, s, NH_2_), 6.25 (1H, s, Ar-H), 6.62 (1H, s, NH), 6.88-6.90 (2H, m, Ar-H_2_), 7.27 (1H, d, *J =* 7.6 Hz, Ar-H), 7.44 (1H, s, Ar-H), 7.66 (1H, s, Ar-H). HR-ESI-MS (m/z): 275.1395 (M+H)^+^ calcd for C_15_H_19_N_2_O_3_ 275.1395.

***tert-*Butyl 2-(4-(chloromethyl)benzamido)-4-(furan-3-yl)phenylcarbamate (4b).** Compound **4a** (500 mg, 1.8 mmol) was treated with *p-*(chloromethyl)benzoyl chloride (411 mg, 2.2 mmol) in the presence of pyridine (0.3 mL) in the same manner as described for the preparation of **3b**, affording **4b** (715 mg, 1.68 mmol, 93%) as pale yellowish crystals. mp: 68-70 °C. ^1^HNMR δ: 1.50 (9H, s, CH_3_ ×3), 4.63 (2H, s, CH_2_), 6.64 - 6.65 (1H, m, Ar-H), 6.86 (1H, s, Ar-H), 7.20-7.28 (2H, m, Ar-H_2_), 7.42-7.49 (3H, m, Ar-H_2_, NH), 7.68 (1H, s, Ar-H), 7.92 (1H, s, Ar-H), 7.97 (2H, B_2_ of A_2_B_2_, *J =* 8.0 Hz, Ar-H_2_), 9.35 (1H, brs, NH). HR-ESI-MS (m/z): 425.1273 (M-H)^+^ calcd for C_23_H_22_ClN_2_O_4_ 425.1268.

***tert-*Butyl 2-(4-((1, 3-dioxoisoindolin-2-yl)methyl)benzamido)-4-(furan-3-yl)phenylcarbamate (4c).** Compound **4b** (500 mg, 1.17 mmol) was reacted with potassium phthalimide (239 mg, 1.3 mmol) and KI (39 mg, 0.24 mmol) in the same manner as described for the preparation of **3c**, yielding pale yellowish crystals **4c** (271 mg, 0.50 mmol, 43%). mp: 97-100°C. ^1^HNMR δ: 1.49 (9H, s, CH_3_ ×3), 4.91 (2H, s, CH_2_), 6.64 (1H, s, Ar-H), 6.83 (1H, s, Ar-H), 7.25 (2H, d, *J =* 8.0 Hz, Ar-H_2_), 7.29-7.31 (1H, m, Ar-H), 7.41-7.45 (1H, m, Ar-H), 7.50 (2H, d, *J =* 8.0 Hz, Ar-H_2_), 7.66-7.68 (1H, m, Ar-H), 7.73 - 7.75 (2H, m, Ar-H_2_), 7.87 (2H, s), 7.92 (2H, d, *J =* 8.0 Hz, Ar-H_2_), 9.19 (1H, brs, NH). HR-ESI-MS (m/z): 536.1819 (M-H)^+^ calcd for C_31_H_26_N_3_O_6_ 536.1822.

***tert-*Butyl 2-(4-(aminomethyl)benzamido)-4-(furan-3-yl)phenylcarbamate (4d).** Compound **4c** (500 mg, 0.93 mmol) was reduced with hydrazine monohydrate (0.09 mL, 2.7 mmol) in the same manner as described for the preparation of **3d**, furnishing **4d** (341 mg, 0.84 mmol, 90%) as pale yellowish crystals. mp: 119-120°C. ^1^HNMR δ: 1.50 (9H, s, CH_3_ ×3), 3.96 (2H, s, CH_2_), 6.64-7.64 (7H, m, Ar-H_6_, NH), 7.88 (2H, s, Ar-H_2_), 7.92 (2H, d, *J =* 8.0 Hz, Ar-H_2_), 9.28 (1H, brs, NH). HR-ESI-MS (m/z): 408.1920 (M+H)^+^ calcd for C_23_H_26_N_3_O_4_ 408.1923.

***tert-*Butyl 2-(4-((4-ethyl-2, 3-dioxopiperazine-1-carboxamido)methyl)benzamido)-4-**

**(furan-3-yl)phenylcarbamate (4e).** Compound **4d** (338 mg, 0.83 mmol) was treated with 4-ethyl-2,3-dioxo-1-piperazinecarbonyl chloride (204 mg, 1.0 mmol) in the presence of Et_3_N (1.5 mL) in the same manner as described for the preparation of **3e**, giving **4e** (259 mg, 0.45 mmol, 54%) as pale yellowish crystals.

mp: 104-105°C. ^1^HNMR δ: 1.23-1.28 (3H, m, CH_3_), 1.50 (9H, s, CH_3_ ×3), 3.53 - 3.58 (4H, m, CH_2_ ×2), 4.09-4.12 (2H, m, CH_2_), 4.58 (2H, d, *J =* 8.0 Hz, CH_2_), 6.65 (1H, s, Ar-H), 6.95 (1H, s, Ar-H), 7.23-7.25 (1H, m, Ar-H), 7.38 (2H, d, *J =* 8.0 Hz, Ar-H_2_), 7.44 (1H, s, Ar-H), 7.67 (1H, s, Ar-H), 7.89 (1H, s, Ar-H), 7.94 (2H, d, *J =* 8.0 Hz, Ar-H_2_), 9.39 (1H, brs, NH), 9.40 (1H, brs, NH). HR-ESI-MS (m/z): 574.2299 (M-H)^+^ calcd for C_30_H_32_N_5_O_7_ 574.2302.

***N-*(4-(2-Amino-5-(furan-3-yl)phenylcarbamoyl)benzyl)-4-ethyl-2, 3-dioxopiperazine-1-carboxamide (4) (K-563).** Compound **4e** (200 mg, 0.35 mmol) was reacted with TFA (2 mL) in the same manner as described for the preparation of **3**, yielding **4** (94 mg, 0.20 mmol, 57%) as pale yellowish crystals.

mp: 175-176°C. ^1^HNMR δ: 1.19 - 124 (3H, m, CH_3_), 3.48 - 3.56 (4H, m, CH_2_ ×2), 3.74 (2H, s, NH_2_), 4.06-4.08 (2H, m, CH_2_), 4.58 (2H, d , *J =* 5.6 Hz, CH_2_), 6.62 (1H, s, Ar-H), 6.84 (1H, d, *J =* 8.0 Hz, Ar-H), 7.26 (1H, s Ar-H), 7.39-7.43 (3H, m, Ar-H_3_), 7.49 (1H, s Ar-H), 7.63 (1H, s Ar-H), 7.88 (2H, d, *J =* 8.0 Hz, Ar-H_2_), 8.08 (1H, s, Ar-H), 9.40 (1H, s, NH).

HR-ESI-MS (m/z): 476.1916 (M+H)^+^ calcd for C_25_H_26_N_5_O_5_ 476.1934.

HPLC purity: >90% (Rt: 7.4 min)

***tert-*Butyl 2-amino-4-(furan-2-yl)phenylcarbamate (5a).** *tert-*Butyl 2-amino-4-bromophenylcarbamate (3.00 g, 10.4 mmol), 2-furanboronic acid (1.51g,13.5 mmol), and *tri-o-*tolylphosphine (1.35g, 4.4 mmol) were treated with K_2_CO_3_ (5.55 g, 40.2 mmol) and tetrakis (triphenyl phosphine) palladium (0.90 g, 0.78 mmol) in the usual manner, giving **5a** (2.3 g, 8.4 mmol, 80%) as pale yellowish crystals. mp: 142-143°C. ^1^H NMR δ: 1.48 (9H, s, CH_3_ ×3), 6.43-6.44 (1H, brs, Ar-H), 6.51 (1H, brs, NH), 6.55 (1H, d, *J* = 3.2 Hz, Ar-H), 7.10-7.11 (2H, m, Ar-H_2_), 7.28-7.31 (1H, m, Ar-H), 7.42 (1H, s, Ar-H); HR-ESI-MS (m/z): 273.1242 (M-H)^+^ calcd. for C_15_H_17_N_2_O_3_ 273.1239.

***tert-*Butyl 2-(4-(chloromethyl)benzamido)-4-(furan-2-yl) phenylcarbamate (5b).** Compound **5a** (500 mg, 1.8 mmol) was treated with *p-*(chloromethyl)benzoyl chloride (410 mg, 2.2 mmol) in the presence of Et_3_N (1.5 mL) in the usual manner, affording **5b** (500 mg, 1.2 mmol, 65%) as pale yellowish crystals. mp: 171-172°C. ^1^HNMR δ: 1.44 (9H, s, CH_3_ ×3), 4.62 (2H, s, CH_2_), 6.42 (1H, d, *J =* 3.2 Hz, Ar-H), 6.54 (1H, d, *J =* 3.2 Hz, Ar-H), 7.26-7.41 (5H, m, Ar-H_4_, NH), 7.45 (2H, A_2_ of A_2_B_2_, *J =* 8 Hz, Ar-H_2_), 7.96 (1H, s, Ar-H), 7.97 (2H, B_2_ of A_2_B_2_, *J =* 8 Hz, Ar-H_2_), 9.51 (1H, brs, NH). HR-ESI-MS (m/z): 425.1278 (M-H)^-^ calcd for C_23_H_22_N_2_O_4_ 425.1268.

***tert-*Butyl 2-(4-((1, 3-dioxoisoindolin-2-yl)methyl)benzamido)-4-(furan-2-yl)phenylcarbamate (5c).** Compound **5b** (500 mg, 1.2 mmol) was treated with potassium phthalimide (239 mg, 1.3 mmol) and KI (39 mg, 0.23 mmol) in the usual manner, yielding **5c** (433 mg, 0.81 mmol, 68%) as colorless crystals. mp: 102-103°C. ^1^HNMR δ: 1.45 (9H, s, CH_3_ ×3), 4.89 (2H, s, CH_2_), 6.36 (1H, brs, Ar-H), 6.50 (1H, brs, Ar-H), 7.33-7.92 (13H, m, Ar-H_12_, NH), 9.48 (1H, brs, NH). HR-ESI-MS (m/z): 536.1817 (M-H)^-^ calcd for C_31_H_26_N_3_O_6_ 536.1822.

***tert-*Butyl 2-(4-(aminomethyl)benzamido)-4-(furan-2-yl)phenylcarbamate (5d).** Compound **5c** (430 mg, 0.80 mmol) was treated with hydrazine monohydrate (0.1 mL, 2.7 mmol) in the usual manner, furnishing **5d** (241 mg, 0.59 mmol, 74%) as pale yellowish crystals.

mp: 171-172°C. ^1^HNMR δ: 1.50 (9H, s, CH_3_ ×3), 3.94 (2H, s, CH_2_), 6.43-8.15 (11H, m, Ar-H_10_, NH), 9.38 (1H, brs, NH). HR-ESI-MS (m/z): 408.1923 (M+H)^+^ calcd for C_23_H_26_N_3_O_4_ 408.1923.

***tert-*Butyl 2-(4-((4-ethyl-2, 3-dioxopiperazine-1-carboxamido)methyl)benzamido)-4-(furan-2-yl)phenylcarbamate (5e).** Compound **5d** (400 mg, 0.98 mmol) was treated with 4-ethyl-2,3-dioxo-1-piperazinecarbonyl chloride (241 mg, 1.2 mmol) in the presence of Et_3_N (1.5 mL) in the usual manner, giving **5e** (466 mg, 0.81 mmol, 83%) as pale yellowish crystals.

mp: 196-198°C. ^1^HNMR δ: 1.22 (3H, t, *J =* 7.0 Hz, CH_3_), 1.49 (9H, s, CH_3_ ×3), 3.46-3.57 (4H, m, CH_2_ ×2), 4.09-4.12 (2H, m, CH_2_), 4.58-4.59 (2H, m, CH_2_), 6.43 (1H, brs, Ar-H), 6.58 (1H, brs, Ar-H), 7.04 (1H, s, Ar-H), 7.04 - 8.14 (8H, m, Ar-H_7_, NH), 9.27 (1H, brs, NH), 9.42 (1H, brs, NH). HR-ESI-MS (m/z): 574.2301 (M-H)^-^ calcd for C_30_H_32_N_5_O_7_ 574.2302.

***N-*(4-(2-Amino-5-(furan-2-yl)phenylcarbamoyl)benzyl)-4-ethyl-2, 3-dioxopiperazine-1-carboxamide (5) (K-564).** Compound **5e** (100 mg, 0.18 mmol) was reacted with TFA (1 mL) in the usual manner, yielding **5** (73 mg, 0.15 mmol, 83%) as pale yellowish crystals. mp: 122-123°C. ^1^HNMR δ: 1.19 (3H, t, *J* = 7.2 Hz, CH_3_), 3.51 (2H, q, *J* = 7.2 Hz, CH_2_), 3.63-3.66 (2H, m, CH_2_), 4.05-4.08 (2H, m, CH_2_), 4.59 (2H, m, CH_2_), 6.45-6.46 (1H, m, Ar-H), 6.59 (1H, d, *J* = 3.2 Hz, Ar-H), 7.00 (1H, d, *J* = 8.0 Hz, Ar-H), 7.45-7.51 (4H, m, Ar-H_4_), 7.58 (1H, s, Ar-H), 7.98 (2H, d, *J* = 8.4 Hz, Ar-H_2_), 9.49 (1H, s, NH). HR-ESI-MS (m/z): 476.1937 (M+H)^+^ calcd for C_25_H_26_N_5_O_5_ 476.1934. HPLC purity: >90% (Rt: 7.7 min)

***tert-*Butyl 2-(4-((4-ethyl-2, 3-dioxopiperazin-1-yl)methyl)benzamido)-**

**4-(thiophen-2-yl) phenylcarbamate (6a).** NaH (170 mg, 60% in mineral oil, 4.2 mmol) was added to a solution of ethyl-2, 3-dioxopiperazine (300 mg, 2.1 mmol) in DMF (5 mL), and the suspension mixture was stirred under ice-cooling for 2 h. After the addition of *tert-*butyl 2-(4-(chloromethyl)benzamido)-4-(thiophen-2-yl)phenylcarbamate (330 mg, 0.74 mmol), the reaction mixture was stirred at RT overnight. The mixture was poured into ice water and extracted with CHCl_3_. The organic layer was successively washed with brine, dried, and concentrated. The resultant product was chromatographed over silica gel with CHCl_3_/MeOH 9:1 as an eluent, yielding colorless crystals **6a** (170 mg, 0.31 mmol, 41.9%).

mp: 119-121°C. ^1^HNMR δ: 1.15 (3H, t, *J =* 6.4 Hz, CH_3_), 1.48 (9H, s, CH_3_ ×3), 3.41-3.50 (6H, m, CH_2_ ×3), 4.66 (2H, d, *J =* 6.4 Hz, CH_2_), 7.00-8.01 (10H, m, Ar-H_10_), 9.71 (1H, brs, NH). HR-ESI-MS (m/z): 549.2173 (M + H)^+^ calcd for C_29_H_33_N_4_O_5_S 549.2171.

***N-*(2-Amino-5-(thiophen-2-yl)phenyl)-4-((4-ethyl-2, 3-dioxopiperazin-1-yl)methyl)benzamide (6) (K-852).** Compound **6a** (50 mg, 0.091 mmol) was dissolved in a mixture of TFA and CH_2_Cl_2_ (1:4) (3 mL). After stirring at RT for 1 h, the solution was successively adjusted to pH 9 with satd. NaHCO_3_, stirred for a further 30 min, washed with brine, dried, and concentrated. The resultant product was recrystallized from EtOH-n-hexane, giving **6** (20 mg, 0.045 mmol, 49.0%) as colorless crystals. mp: 130-131°C. ^1^HNMR δ: 1.14 (3H, t, *J =* 6.4 Hz, CH_3_), 3.41-3.50 (6H, m, CH_2_ ×3), 4.66 (2H, d, *J =* 6.4 Hz, CH_2_), 7.00-7.61 (10H, m, Ar-H_10_), 7.94 (1H, brs, NH). HR-ESI-MS (m/z): 449.1660 (M + H)^+^ calcd for C_24_H_25_N_4_O_5_S 449.1647. HPLC purity: >90% (Rt: 7.2 min).

***tert-*Butyl 2-(4-(((*S*)-1,4-dioxo-hexahydropyrrolo[1,2-a]pyrazin-2(1H)-yl)methyl)-**

**benzamido)-4-(thiophen-2-yl)phenylcarbamate (7a).** A solution of cyclo-L-prolylglycine (300 mg, 2.11 mmol) in DMF (5 mL) was treated with *tert-*butyl 2-(4-(chloromethyl)benzamido)-4-(thiophen-2-yl) phenylcarbamate (350 mg, 0.70 mmol) in the presence of NaH (170 mg, 60% in mineral oil, 4.2 mmol) in the same manner as described for the preparation of **6a**. The resultant product was chromatographed with EtOAc/n-hexane 1:4 and then with CHCl_3_/MeOH 9:1 as eluents, yielding **7a** (120 mg, 0.21 mmol, 30.0%) as colorless crystals. mp: 113-114°C. ^1^HNMR δ: 1.49, 1.50, 1.52 (9H, each s, CH_3_ ×3), 1.87-2.48 (4H, m, CH_2_ ×2), 3.52-3.78 (3H, m, -CH-×3), 4.00 (1H, d, *J =* 16.4 Hz, -CH-), 4.15 (1H, t, *J =* 7.2 Hz, -CH-), 4.49 (1H, d, *J =* 14.8 Hz, -CH-) 4.80 (1H, d, *J =* 14.8 Hz, -CH-), 7.14-7.41 (6H, m, Ar-H_6_), 7.89-7.97 (3H, m, Ar-H_3_), 9.40 (1H, brs, NH). HR-ESI-MS (m/z): 561.2178 (M + H)^+^ calcd for C_30_H_33_N_4_O_5_S 561.2171.

***N-*(2-Amino-5-(thiophen-2-yl)phenyl)-4-(((*S*)-1,4-dioxo-hexahydropyrrolo[1,2-a]pyrazin-2(1H)-yl)methyl)benzamide (7) (K-854).** Compound **7a** (100 mg, 0.17 mmol) was treated with a mixture of TFA and CH_2_Cl_2_ (1:4) (3 mL) in the same manner as described for the preparation of **6**. The product was purified by chromatography over silica gel with CHCl_3_/MeOH 9:1 as an eluent and recrystallization from CHCl_3_/n-hexane, yielding **7** (30 mg, 0.065 mmol, 38.2%) as colorless crystals. mp: 187-190°C. ^1^HNMR δ: 1.90, 2.23 (4H, each m, CH_2_ ×2), 3.69 (1H, d, *J =* 16.4 Hz, -CH-), 4.17 (1H, d, *J =* 16.8 Hz, -CH-), 4.31 (1H, t, *J =* 7.2 Hz, -CH-), 4.63 (2H, s, -NH_2_), 5.17 (2H, s, CH_2_), 6.74-7.50 (7H, m, Ar-H_7_), 7.96 (2H, m, Ar-H_2_), 9.68 (1H, m, NH). HR-ESI-MS (m/z): 461.1645 (M + H)^+^ calcd for C_25_H_25_N_4_O_3_S 461.1647. HPLC purity: >90% (Rt: 7.7 min)

***tert-*Butyl 2-(4-((4-methyl-2, 5-dioxopiperazin-1-yl)methyl)benzamido)-4-**

**(thiophen-2-yl)phenylcarbamate (8a).** A solution of 1-methylpiperazine-2,5-dione (100 mg, 0.78 mmol) in DMF (5 mL) was reacted with *tert-*butyl 2-(4-(chloromethyl)benzamido)-4-(thiophen-2-yl)phenylcarbamate (350 mg, 0.78 mmol) in the presence of NaH (75 mg, 60% in mineral oil, 1.9 mmol) in the usual manner. The resultant product was chromatographed over silica gel with CHCl_3_./MeOH 9:1, yielding pale yellowish crystals **8a** (178 mg, 0.33 mmol, 42.7%). mp: 110-112°C. ^1^HNMR δ: 1.51 (9H, brs, CH_3_ ×3), 2.99 (3H, s, CH_3_), 3.88 (2H, s, -COCH_2_-), 4.07 (2H, s, -COCH_2_-), 4.64 (2H, brs, -CH_2_-), 7.00-7.98 (10H, m, Ar-H), 9.38 (1H, s, NH). HR-ESI-MS (m/z): 535.2013 (M + H)^+^ calcd for C_28_H_31_N_4_O_5_S 535.2016.

***N-*(2-Amino-5-(thiophen-2-yl)phenyl)-4-((4-methyl-2, 5-dioxopiperazin-1**

**-yl)methyl)benzamide (8) (K-856).** Compound **8a** (30 mg, 0.056 mmol) was dissolved in a mixture of TFA and CH_2_Cl_2_ (1:4) (3 mL). After stirring at RT for 3 h, the solution was treated in the usual manner. The resultant product was recrystallized from EtOH, giving **8** (14 mg, 0.031 mmol, 55%) as colorless crystals. mp: 214-216°C. ^1^H NMR ((CD_3_)_2_SO) δ: 2.84 (3H, brs, CH_3_), 3.87 (2H, s, -COCH_2_-), 4.07 (2H, s, -COCH_2_-), 4.61 (2H, s, CH_2_), 5.20 (1H, brs, NH), 6.72-7.50 (8H, m, Ar-H_8_), 7.94-8.32 (2H, m, Ar-H_2_), 9.74 (1H, brs, NH). HR-ESI-MS (m/z): 435.1501 (M + H)^+^ calcd for C_23_H_23_N_4_O_3_S 435.1492. HPLC purity: >90% (Rt: 7.4 min).

***N-*(2-Amino-5-(thiophen-2-yl)phenyl)-4-((4-methyl-3-oxopiperazin-1**

**-yl)methyl)benzamide (9) (OP-857).** A solution of 1-methylpiperazin-2-one (0.24 mL, 2.20 mmol) in THF (5 mL) was reacted with *tert-*butyl 2-(4-(chloromethyl)benzamido)-4-(thiophen-2-yl)phenylcarbamate (200 mg, 0.45 mmol) in the presence of NaH (200 mg, 60% in mineral oil, 5.0 mmol) in the usual way, yielding a crude powder **9a** (55 mg). An aliquot (22 mg) of **9a** was Boc-deblocked with a mixture of TFA and CH_2_Cl_2_ (1:4) (3 mL) in the usual way. Silica gel chromatography and PLC of the resultant product with CHCl_3_/MeOH 9:1 yielded **9** (7 mg) as a solid. mp: 115-117°C. ^1^HNMR δ: 3.50 (2H, brs, CH_2_), 3.64 (2H, s, N-CH_3_), 3.90 (2H, brs, CH_2_), 4.92 (2H, s, CH_2_), 5.09 (2H, brs, CH_2_), 7.00-7.9 (10H, m, Ar-H_10_), 8.96 (1H, brs, NH). HR-ESI-MS (m/z): 421.1689 (M + H)^+^ calcd for C_23_H_25_N_4_O_2_S 421.1699. HPLC purity: >90% (Rt: 7.4 min).

***N-*(2-Amino-5-(thiophen-2-yl)phenyl)-4-((4-methyl-3-oxopiperazin-1-methyl)benzamide (10) (OP-858).** A solution of *tert-*butyl 3-oxopiperazine-1-carboxylate (200 mg, 1.0 mmol) in THF (5 mL) was treated with *tert-*butyl 2-(4-(chloromethyl)benzamido)-4-(thiophen-2-yl)phenylcarbamate (200 mg, 0.45 mmol) in the presence of NaH (200 mg, 60% in mineral oil, 5.0 mmol), yielding a crude powder **10a** (30 mg). An aliquot (20 mg) of **10a** was treated with a mixture of TFA and CH_2_Cl_2_ (1:4) (3 mL). The product was purified in the same manner as described for the purification of **9**, giving **10** (6 mg) as a white powder. mp: 154-155°C. ^1^HNMR δ: 3.38 (2H, brs, CH_2_), 3.63 (2H, brs, CH_2_), 4.09 (2H, s, CH_2_), 4.91 (2H, brs, CH_2_), 6.79-7.96 (10H, m, Ar-H_10_), 9.18 (1H, brs, NH). HR-ESI-MS (m/z): 407.1546 (M + H)^+^ calcd for C_22_H_23_N_4_O_2_S 407.1543. HPLC purity: >90% (Rt: 3.2 min).

***N-*(2-Amino-5-(thiophen-2-yl)phenyl)-4-((4-ethylpiperazin-1**

**-yl)methyl)benzamide (11) (OP-859).** A solution of 1-ethylpiperazine (0.2 mL, 1.95 mmol) in THF (5 mL) was reacted with *tert-*butyl 2-(4-(chloromethyl)benzamido)-4-(thiophen-2-yl)phenylcarbamate (200 mg, 0.45 mmol) in the presence of NaH (200 mg, 60% in mineral oil, 5.0 mmol), giving rise to **11a** as a crude powder (42 mg). An aliquot (20 mg) of **11a** was Boc-deblocked with a mixture of TFA and CH_2_Cl_2_ (1:4) (3 mL). Silica gel chromatography and PLC of the resultant product with CHCl_3_/MeOH 8:2 gave **11** (9 mg) as a solid. mp: 160-161°C. ^1^HNMR δ: 1.09 (3H, t, *J =* 7.0 Hz, CH_3_), 2.39-2.43 (6H, m, CH_2_ ×3), 2.90-2.93 (4H, m, CH_2_ ×2), 4.91 (2H, s, CH_2_), 7.10-8.00 (10H, m, Ar-H_10_), 7.94 (1H, brs, NH). HR-ESI-MS (m/z): 421.2065 (M + H)^+^ calcd for C_24_H_29_N_4_OS 421.2063. HPLC purity: >90% (Rt: 11.8 min).
